# Supplementary material for: Regional Transmission and Reassortment of 2.3.4.4b Highly Pathogenic Avian Influenza (HPAI) Viruses in Bulgarian Poultry 2017/18
Source: Viruses. 2020 Jun 1;12(6):605. doi: 10.3390/v12060605 (PMC7354578; doi:10.3390/v12060605)
Supplement: Supplementary file 1 [file viruses-12-00605-s001.pdf]

viruses

MDPI

Supplementary Figures

Regional Transmission and Reassortment of 2.3.4.4b Highly Pathogenic Avian Influenza (HPAI) Viruses in Bulgarian Poultry 2017/18

Divya Venkatesh [1,*], Adam Brouwer [2], Gabriela Goujgoulova [3], Richard Ellis [4], James Seekings [2,5], Ian H. Brown [2] and Nicola S. Lewis [1,2]

[1] Department of Pathobiology and Population Sciences, Royal Veterinary College, Hatfield, Hertfordshire, AL9 7TA, United Kingdom; nilewis@rvc.ac.uk
[2] OIE/FAO/ International Reference Laboratory for avian influenza, swine influenza and Newcastle Disease, Animal and Plant Health Agency (APHA), Weybridge, Addlestone, Surrey, KT15 3NB, United Kingdom; adam.brouwer@apha.gov.uk (A.B.); james.seekings@apha.gov.uk (J.S.), ian.brown@apha.gov.uk (I.H.B.)
[3] National Diagnostic Research Veterinary Medical Institute, Sofia, 1231, Bulgaria; gvgoujgoulova@abv.bg
[4] Surveillance and Laboratory Services Department, Animal and Plant Health Agency (APHA), Weybridge, Addlestone, Surrey, KT15 3NB, United Kingdom; Richard.Ellis@apha.gov.uk
[5] Virology Department, Animal and Plant Health Agency (APHA), Weybridge, Addlestone, Surrey, KT15 3NB, United Kingdom
* Correspondence: dvenkatesh@rvc.ac.uk

Received: 27 April 2020; Accepted: 28 May 2020; Published: date

Abstract: Between 2017 and 2018, several farms across Bulgaria reported outbreaks of H5 highly-pathogenic avian influenza (HPAI) viruses. In this study we used genomic and traditional epidemiological analyses to trace the origin and subsequent spread of these outbreaks within Bulgaria. Both methods indicate two separate incursions, one restricted to the northeastern region of Dobrich, and another largely restricted to Central and Eastern Bulgaria including places such as Plovdiv, Sliven and Stara Zagora, as well as one virus from the Western region of Vidin. Both outbreaks likely originate from different European 2.3.4.4b virus ancestors circulating in 2017. The viruses were likely introduced by wild birds or poultry trade links in 2017 and have continued to circulate, but due to lack of contemporaneous sampling and sequences from wild bird viruses in Bulgaria, the precise route and timing of introduction cannot be determined. Analysis of whole genomes indicates a complete lack of reassortment in all segments but the matrix protein gene (MP), which presents as multiple smaller clusters associated with different European 2.3.4.4b viruses. Ancestral reconstruction of host states of the hemagglutinin (HA) gene of viruses involved in the outbreaks suggests that transmission is driven by domestic ducks into galliform poultry. Thus, according to present evidence, we suggest the surveillance of domestic ducks as they are an epidemiologically relevant species for subclinical infection. Monitoring the spread due to movement between farms within regions and links to poultry production systems in European countries can help to predict and prevent future outbreaks. The 2.3.4.4b lineage which caused the largest recorded poultry epidemic in Europe continues to circulate, and the risk of further transmission by wild birds during migration remains.

Keywords: avian influenza; HPAI (highly pathogenic avian influenza); 2.3.4.4b; poultry outbreak; genomic epidemiology

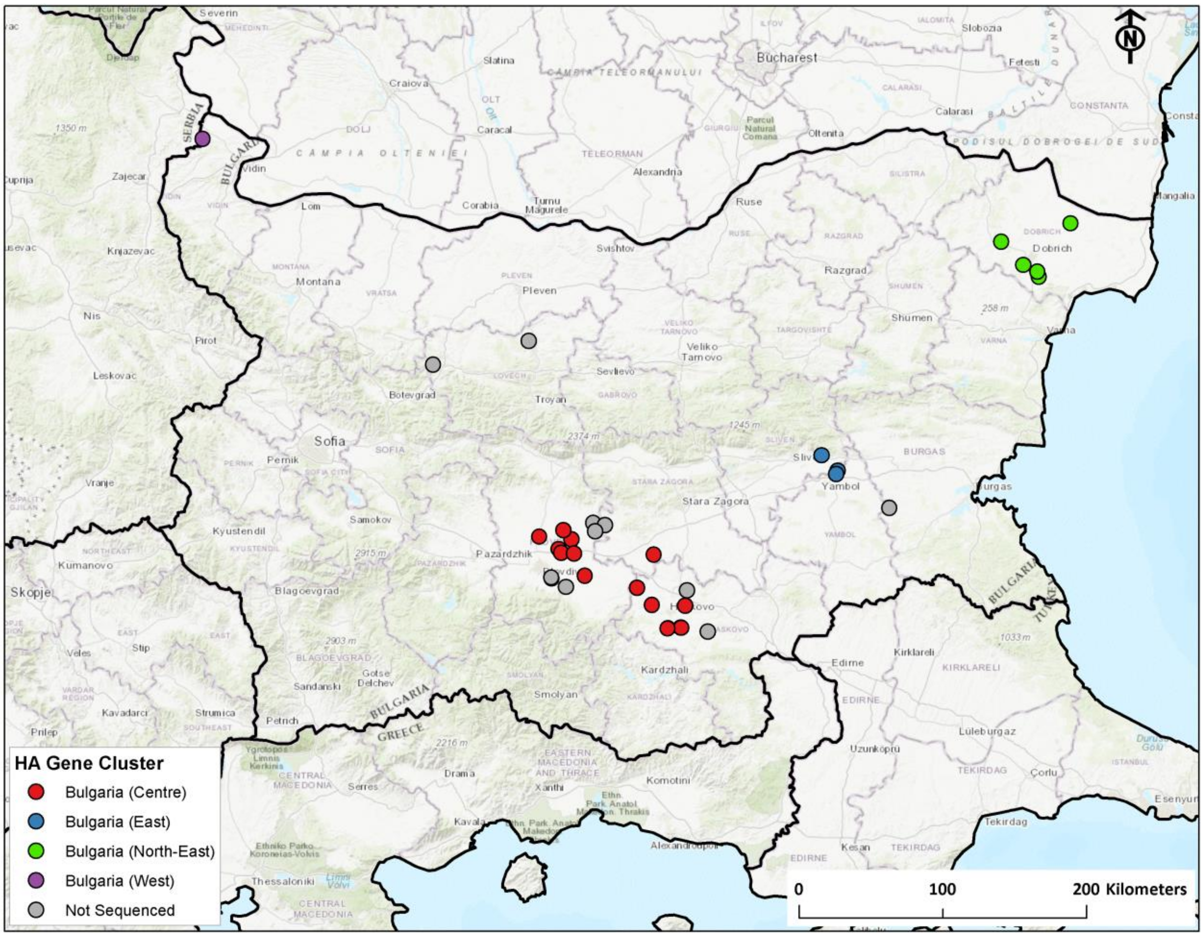

Supplementary Figure S1: Map showing geographical distribution of viral genetic clusters in Bulgaria between 2017–2018.

Supplementary Table S1: Wild Bird Surveillance results from Bulgaria, Romania and Greece (2016–2018). HPAI: Highly pathogenic avian influenza

	2016			2017			2018		
**Country**	**Number of birds sampled**	**Total number of HPAI H5 detections**	**Total number of HPAI H5N8 detections**	**Number of birds sampled**	**Total number of HPAI H5 detections**	Total number of HPAI H5N8 detections	**Number of birds sampled**	**Total number of HPAI H5 detections**	**Total number of HPAI H5N8 detections**
Bulgaria	9	2	2	47	14	14	58	0	0
Greece	16	1	1	90	13	12	13	0	0
Romania	275	10	10	528	162	143	244	0	0

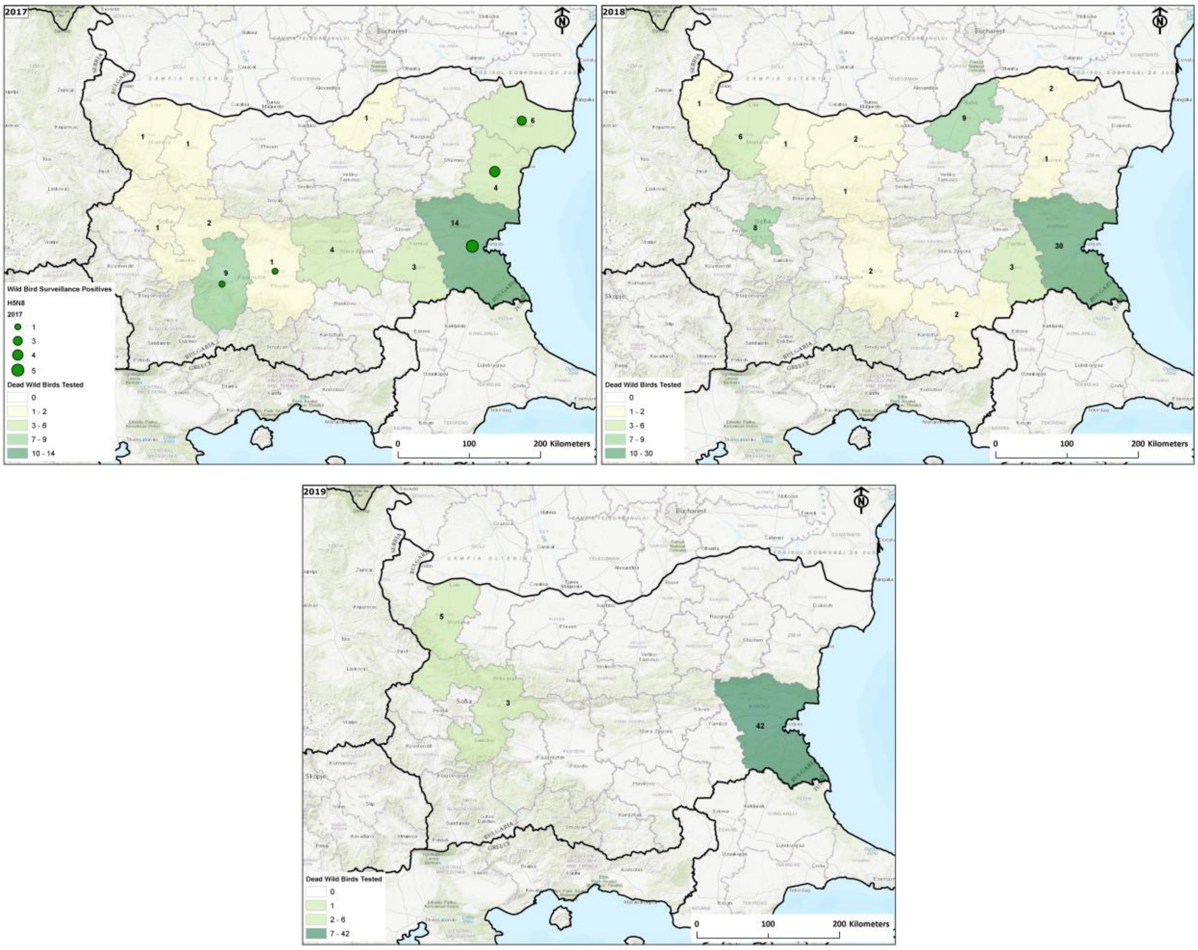

Supplementary Figure S2: Clockwise from top left: Surveillance of dead wild birds in Bulgaria from 2017, 2018 and 2019 (first six months). Green circles represent H5N8 wild bird positives, regional shading indicates number of dead wild birds tested

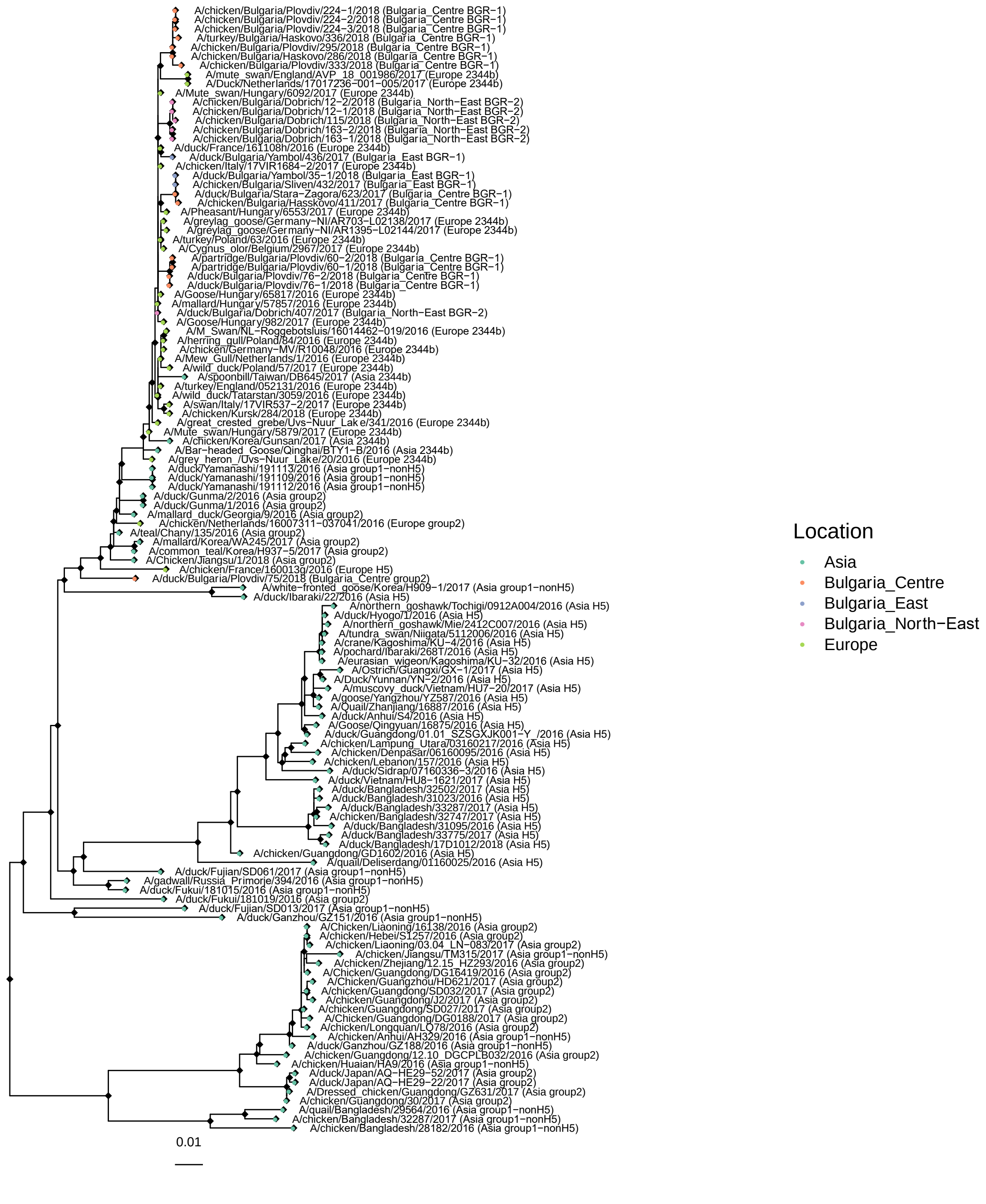

Supplementary Figure S3: Maximum-likelihood tree for MP (matrix protein) gene segment from Bulgarian (2017–18) and related viruses. Colours indicate location the virus was isolated from, and black diamonds at nodes indicate approximate likelihood ratio test (aLRT) branch supports > 0.9.

Supplementary Table S2: A record of highly pathogenic avian influenza disease events recorded in Bulgaria between 1 January 2019 and 8 April 2020. Table downloaded on 8 April 2020 from the FAO (Food and Agriculture Organisation) EMPRES-i (EMPRES Global Animal Disease Information System) initiative website: http://empres-i.fao.org/eipws3g/.

Id	source	region	country	admin1	locality Name	observation Date	reporting Date	status	disease	serotypes	species Description	sum AtRisk	sum Cases	sum Deaths	sum Destroyed
266680	EC	Europe	Bulgaria	Plovdiv	Trilistnik village	12/03/2020	13/03/2020	Confirmed	Influenza - Avian	H5N8 HPAI	domestic, chicken	39120	50		
266679	EC	Europe	Bulgaria	Kardzhal	Kurdzhali municipal	11/03/2020	13/03/2020	Confirmed	Influenza - Avian	H5N8 HPAI	domestic, chicken	16800	8000		
266161	OIE	Europe	Bulgaria	Plovdiv	Bulgaria	02/03/2020	03/03/2020	Confirmed	Influenza - Avian	H5N8 HPAI	domestic, unspecified bi	5000	20		5000
266160	OIE	Europe	Bulgaria	Plovdiv	Bulgaria	02/03/2020	03/03/2020	Confirmed	Influenza - Avian	H5N8 HPAI	domestic, unspecified bi	3620	20		3620
265627	OIE	Europe	Bulgaria	Plovdiv	Bulgaria	24/02/2020	24/02/2020	Confirmed	Influenza - Avian	H5N8 HPAI	domestic, duck	11600	20	20	11580
265626	OIE	Europe	Bulgaria	Plovdiv	Bulgaria	24/02/2020	24/02/2020	Confirmed	Influenza - Avian	H5N8 HPAI	domestic, chicken	55437	34	34	55403
265136	National authorities	Europe	Bulgaria	Plovdiv	Bulgaria	17/02/2020	17/02/2020	Confirmed	Influenza - Avian	H5N8 HPAI	domestic, duck	15729	9142	9142	5830
250626	National authorities	Europe	Bulgaria	Plovdiv	Asenovgrad	08/04/2019	08/04/2019	Confirmed	Influenza - Avian	H5N8 HPAI	domestic, chicken	168752	300	300	168452
250532	National authorities	Europe	Bulgaria	Plovdiv	Krumovo	03/04/2019	05/04/2019	Confirmed	Influenza - Avian	H5N8 HPAI	domestic, chicken	37	37	37	0
250531	National authorities	Europe	Bulgaria	Plovdiv	Asenovgrad	03/04/2019	05/04/2019	Confirmed	Influenza - Avian	H5N8 HPAI	domestic, chicken	34998	298	298	34700
250202	OIE	Europe	Bulgaria	Lovech	Lovech	02/04/2019	03/04/2019	Confirmed	Influenza - Avian	H5 HPAI	domestic, unspecified bi	1400	20	0	1400
248811	OIE	Europe	Bulgaria	Lovech	Lisets	13/03/2019	13/03/2019	Confirmed	Influenza - Avian	H5 HPAI	domestic, unspecified bi	3200	20	0	
